# Known Antimicrobials Versus Nortriptyline in *Candida albicans*: Repositioning an Old Drug for New Targets

**DOI:** 10.3390/microorganisms8050742

**Published:** 2020-05-15

**Authors:** Marina Caldara, Nelson Marmiroli

**Affiliations:** 1 Department of Chemistry, Life Sciences and Environmental Sustainability, University of Parma, Parco Area delle Scienze 11/A, 43124 Parma, Italy; nelson.marmiroli@unipr.it; 2 Interdepartmental Center SITEIA.PARMA, University of Parma, Parco Area delle Scienze 181/A, 43124 Parma, Italy

**Keywords:** *Candida albicans*, antimicrobial agents, nortriptyline, drug transporters

## Abstract

*Candida albicans* has the capacity to develop resistance to commonly used antimicrobials, and to solve this problem, drug repositioning and new drug combinations are being studied. Nortriptyline, a tricyclic antidepressant, was shown to have the capacity to inhibit biofilm and hyphae formation, along with the ability to efficiently kill cells in a mature biofilm. To use nortriptyline as a new antimicrobial, or in combination with known drugs to increase their actions, it is important to characterize in more detail the effects of this drug on the target species. In this study, the *Candida albicans* GRACE™ collection and a Haplo insufficiency profiling were employed to identify the potential targets of nortriptyline, and to classify, in a parallel screening with amphotericin B, caspofungin, and fluconazole, general multi-drug resistance genes. The results identified mutants that, during biofilm formation and upon treatment of a mature biofilm, are sensitive or tolerant to nortriptyline, or to general drug treatments. Gene ontology analysis recognized the categories of ribosome biogenesis and spliceosome as enriched upon treatment with the tricyclic antidepressant, while mutants in oxidative stress response and general stress response were commonly retrieved upon treatment with any other drug. The data presented suggest that nortriptyline can be considered a “new” antimicrobial drug with large potential for application to in vivo infection models.

## 1. Introduction

*Candida* spp. are one of the most frequent nosocomial pathogens often causing bloodstream infections [1], and with the capacity to develop resistance to commonly used antifungals. To fight this problem, drug repurposing [2,3] along with the identification of new active molecules and new combination strategies [4,5] are being investigated. Thanks to drug repositioning, ibuprofen has recently been proven to be efficient against *Staphylococcus aureus* biofilms [6], while auranofin has been shown to be a strong antimicrobial [7]. Moreover, sertraline, an antidepressant inhibiting the selective reuptake of serotonin, was shown to induce autophagy in *Saccharomyces cerevisiae* [8], and its antifungal activity is now being evaluated with in vivo infection models of *Aspergillus fumigatus* [9] and *Cryptococcus neoformans* [10]. Nortriptyline (NOR), the most used tricyclic antidepressants (TCA) up until the introduction of selective serotonin re-uptake inhibitors, or SSRIs, was shown to have the capacity to inhibit biofilm and hyphae formation and to efficiently kill cells in a mature biofilm of *C. albicans* [11]. NOR induces lysis of cells [11], an effect also displayed upon treatment with local anesthetics [12] and antipsychotic phenothiazine [13]. This phenomenon is related to their amphiphilic structures and their surfactant activity, which are able to disturb cell membrane functionality [11,13]. Interestingly, NOR is now also employed in clinical trials focused not only on depression, but also on atopic dermatitis, psoriasis vulgaris, irritable bowel syndrome, and gastro-esophageal reflux disease (www.drugbank.ca/drugs/DB00540).

In order to propose NOR as a new antimicrobial, on its own or in combination with known antimicrobials to potentiate their actions, it is important to characterize in more detail the effects of this drug on the pathogenic yeast *Candida albicans*. To do this, the Haplo insufficiency profiling [14] (HIP approach) could be used. This procedure is based on the assumption that a heterozygous deletion strain would be particularly sensitive to the presence of a drug whenever the product of the deleted gene is the target of the molecule being tested. Binding will lower its activity, giving rise to a more sensitive or tolerant phenotype depending on the biological role carried out by the target [15]. With this strategy, new drug actions and novel gene functions have already been reported [16,17]. When needed, the HIP approach can also be combined with homozygous profiling assay (HOP), where complete loss-of-function is tested.

In this work, the *Candida albicans* GRACE™ collection and a HIP approach were used to identify the potential targets of NOR by comparing its action with that of three known antimicrobials: amphotericin B (AMB), caspofungin (CAS), and fluconazole (FLU), in order to classify NOR specific targets and general multi-drug resistance (MDR) genes. In addition, tests on oxidative stress, mutagenicity, and expression of drug transporters were performed.

## 2. Materials and Methods 

### 2.1. Strains and Media

The *C. albicans* GRACE™ mutant collection (2372 strains) [18] was obtained in the CaSS1 background by replacing one copy of the target gene with a cassette containing the selectable marker *HIS3* flanked by the necessary homologous sequences and two distinct bar codes, and by swapping the promoter region of the second copy of the gene with a cassette containing the tightly regulated tetracycline promoter, which is repressed when the media is supplemented with tetracycline. In this screening, no tetracycline was employed (except for selected mutants reported in Supplementary Figure S1), allowing the expression of one active copy of the gene of interest and the employment of HIP assay [16]. Strains were grown either in YPD (Yeast extract-Peptone- Dextrose) or in RPMI-1640 (Roswell Park Memorial Institute-1640) (Thermo Fisher Scientific Inc., Waltham, MA, USA) with 2% glucose as specified. Bacteria strains *S.typhimurium* TA98 and TA100 were kindly provided by Prof. R. Scarpato (Dep. of Biology, University of Pisa, Italy) and grown in Nutrient Broth Oxoide no.2 (Thermo Fisher Scientific Inc., Waltham, MA, USA).

### 2.2. Testing Drug Working Concentrations

The first step was to identify a concentration for each drug that allowed a clear identification of the growth phenotype (GP) displayed by the mutants. Specifically, concentrations that allowed growth of wild type (WT), but not of sensitive mutants (condition A), and concentrations where the WT could grow poorly, but that allow the growth of tolerant mutants (condition B). With respect to condition A, NOR, AMB, CAS, and FLU were used at 10 µM, 0.5 µM, 15 µM, and 200 µM, respectively, while in condition B, they were used at 20 µM, 1 µM, 20 µM, and 400 µM, respectively. Chemicals were purchased at Sigma-Aldrich-Merck KGaA (Darmstadt, Germany) unless specified otherwise.

### 2.3. Identification of Mutants Sensitive or Tolerant to Drugs during Biofilm Formation

Mutants were pre-grown in YPD, and cells were then transferred with a 96-multi pins apparatus to plates containing liquid RPMI-1640 supplemented with 2% glucose and maintained for 90 min at 37 °C at 150 rpm. At the end, the media was removed and the adherent cells were incubated for 48 h in the same media containing NOR (or the other specific drugs of interest), which has a half-life of 37 h [19]. Each plate was grown in triplicate and compared to a plate where no antifungals were added. At the end of the incubation, the GP was evaluated with spectrophotometric reading. A mutant was considered sensitive when its growth was at least one-third of that displayed by the WT and tolerant when the GP was at least three times that of the WT.

### 2.4. Identification of Mutants Sensitive or Tolerant to Drugs after Biofilm Growth

Mutants were grown as described in the previous paragraph, but at the end of the 90 min of incubation, adherent cells were incubated in RPMI-1640 with 2% glucose for 48 h at 37 °C and 150 rpm. Then, the exhausted medium was removed, and the biofilm was treated with fresh medium containing the drug of interest for a further 48 h. Growth and vitality were evaluated using the XTT (2,3-Bis-(2-Methoxy-4-Nitro-5-Sulfophenyl)-2H-Tetrazolium-5-Carboxanilide) reduction assay, as described previously [11].

### 2.5. Glutathione and Nitric Oxide Assays

Cells were grown in RPMI-1640 with 2% glucose with or without the presence of drugs at the sub-inhibitory concentration MIC_75_ (corresponding to 75% of the MIC_99_) for 24 h at 37 °C with shaking, after which 10^7^ cells were collected and lysed. Glutathione oxidized/reduced forms were assessed by measuring the formation of 2-nitro-5-thiobenzoate, while the nitric oxide (NO) non-enzymatic assay kit (Oxford Biomedical Research, Rochester Hills, MI, USA) was used to determine the concentration of NO, as previously described [20]. Colorimetric reactions were assayed using an iMARK microplate absorbance reader (Bio-Rad, Hercules, CA, USA).

### 2.6. Measurements of Reactive Oxygen Species (ROS) and Cells Viability by Flow Cytometry

For ROS measurements, freshly grown cells were diluted out to a concentration of 10^6^ cells mL^−1^ and incubated with 20 µM of 2′7′-dichlorofluorescein diacetate for 30 min at 37 °C. Cells were then collected and resuspended in fresh media with or without the drugs at their MIC_75_ and incubated for 4 h. The percentage of the population presenting 2′7′-dichlorofluorescein, thus containing ROS (and identified as ROS+), was assayed by flow cytometry (NovoCyte; ACEA Biosciences, Inc., San Diego, CA, USA) in parallel with the estimation of the percentage of dead cells incorporating propidium iodide (PI); both procedures were previously described [20]. The experiment was performed in triplicate and, for each condition, 12,000 cells were analyzed.

### 2.7. Isolation of RNA and Quantitative Reverse Transcription Polymerase Chain Reaction (qRT-PCR)

Gene expression was evaluated in a mature biofilm grown for 48 h and incubated with the MIC_75_ of each molecule of interest for 4 or 24 h. Extractions of total RNA, retrotranscriptions, qRT-PCR, and relative quantification were performed as previously described [11]. The primers employed are listed in Supplementary Table S1.

### 2.8. Ames Test

Mutagenicity test was performed as described previously [21] with few modifications: 100 µL of freshly grown bacteria (Optical Density OD_660_ = 0.3) was incubated for 30 min at 37 °C in the presence of NOR (60 µg and 12µg) or the known mutagenic substances 2-nitrofluorene (2 µg) or 2-aminoantracene (1 µg) with or without 500 µL of S9 (S9 SD rat liver Aroclor in KCl, Trinova Biochem Gmbh) mixture (phosphate buffer, 4mM NADP (Nicotinamide adenine dinucleotide phosphate), 5 mM glucose-6-phosphate, 33 mM MgCl_2_, 8 mM KCl, S9 fraction 4%) in phosphate buffer (12.5 mM Na_2_HPO_4_ and 100 mM NaH_2_PO_4_·H_2_O). The negative control was done by incubating cells in phosphate buffer without any drugs. After the incubation, 1.3 mL of soft agar (0.6% w/vol) containing histidine and biotin (0.5 mM) was added to the Eppendorf and dispensed on solid MGA (Minimal Glucose Agar) medium (0.53% potassium phosphate (dibasic), 0.2% potassium phosphate monobasic, 0.1% ammonium sulfate, 0.05% sodium citrate, dehydrate, 0.4% glucose, 1.6% agar). Revertant colonies were counted after three days of incubation at 37 °C.

### 2.9. Statistical and Bioinformatics Analysis

Data analysis was performed using SPSS (IBM Corp., Armonk, NY, USA). Network was obtained using STRING and the *Candida albicans* dataset, allowing interaction with at least medium confidence (0.4) and displaying a maximum of 10 nodes from the first and second shell of interactions. The interactions displayed are evidence-based. Disconnected nodes were removed from the analysis. Average local clustering coefficients were 0.734 (enriched categories for NOR sensitive mutants during biofilm formation), 0.508 (enriched categories for NOR sensitive mutants in a mature biofilm), 0.527 (enriched categories for NOR tolerant mutants during biofilm formation), and 0.605 (enriched categories for NOR tolerant mutants in a mature biofilm).

## 3. Results

### 3.1. Determination of GPs: Identification of Mutants Sensitive to NOR

**Figure 1:**
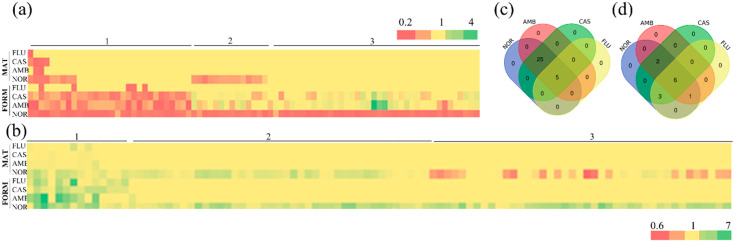
Identification of sensitive and tolerant mutants: heat maps of sensitive (**a**) and tolerant (**b**) mutants identified in the screening based on the growth phenotypes (GPs). Values of the GPs ratios (mutant vs. wild type control) recorded in the screening are reported at the bottom of the map. Each row represents a drug treatment (nortriptyline (NOR), amphotericin B (AMB), caspofungin (CAS), and fluconazole (FLU)) during biofilm formation (FORM) or on a mature biofilm (MAT). Three groups were identified: mutants sensitive or tolerant to more than one drug (1), sensitive and tolerant to NOR during biofilm formation or maturation (2), and sensitive and tolerant only during biofilm formation (3). The Venn diagrams presented in (**c**) and (**d**) analyze in more detail the overlap between the given GPs upon drug treatments of sensitive or tolerant mutants.

The screening of the GRACE™ library allowed to identify mutants with a different GP when grown in the presence of NOR. These heterozygotes mutants express only one functional copy of a specific gene and, following the HIP approach, become more sensitive to any drug acting on the product of the gene [15]. Therefore, this type of study could help in identifying the target of the drug that is being investigated. With a triplicate screening of the library, a list of strains sensitive to NOR was compiled (Figure 1a, Supplementary Table S2). The displayed GPs allow the separation of the mutants into three categories: the first one corresponds to mutants sensitive to NOR, as well as to other known antimicrobials and in all the growing conditions tested; the second one corresponds to mutants sensitive when NOR was added during the first stages of biofilm formation and to the mature biofilm; and the third corresponds to strains sensitive to NOR only during the first stages of biofilm formation (Figure 1a).

**Figure 2:**
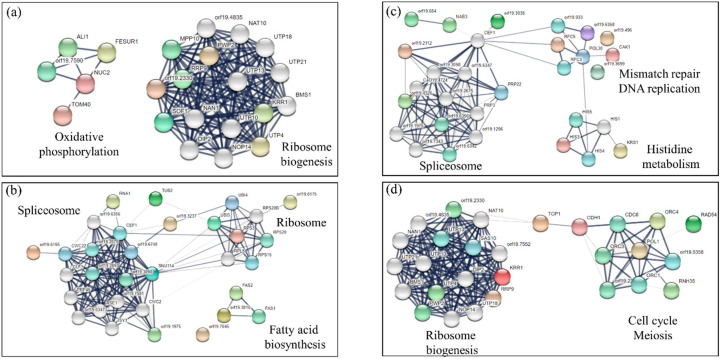
Network obtained with STRING of the enriched functional categories that, when modified in *Candida albicans*, gave a sensitive GP upon treatment with NOR during biofilm formation (**a**) or when grown in a mature biofilm (**b**), or a tolerant GP upon treatment with NOR during biofilm formation (**c**) or even when grown in a mature biofilm (**d**).

The third category includes 15 mutants, most of which are poorly characterized. Considering the networks generated by these knock-out genes, and their first interactors, an enrichment of the KEGG (Kyoto Encyclopedia of Genes and Genomes) pathway’s (medium confidence and False Discovery Rate FDR ˂ 0.005) ribosome biogenesis and oxidative phosphorylation was observed (Figure 2a). When the expression of the second copy of the gene was also repressed in selected mutants (*cdc19*Δ*/cdc19*Δ, *mlh3*Δ*/mlh3*Δ, *spt8*Δ*/spt8*Δ) by adding tetracycline, the identified mutants became sensitive to NOR even in a mature biofilm (Supplementary Figure S1). A total of 38 mutants were identified as sensitive to the TCA also within a mature biofilm. Network analysis for these revealed an enrichment in genes related to spliceosome activity, ribosome machinery, and fatty acid biosynthesis (Figure 2b). For the sensitive mutants, the enriched categories identified with Gene Ontology (GO) Process Slim Mapper (Supplementary Table S3 were RNA metabolism, transport, organelle organization, ribosome biogenesis, cellular protein modification, and response to chemicals.

### 3.2. Identification of Mutants Tolerant to NOR

The same type of analysis also produced evidence on mutants more tolerant to the presence of NOR. As shown in Supplementary Table S4 and Figure 1b, 44 strains grew better than the WT when challenged with the TCA during biofilm formation. The spliceosome system, mismatch repair/DNA replication, and histidine metabolism were the enriched GO categories (Figure 2c). Inactivating the second copy of the gene as well (*pmu5*Δ*/pmu5*Δ, *dal9*Δ*/dal9*Δ, *pet127*Δ*/pet127*Δ) by adding tetracycline to the growth medium makes the selected mutants more tolerant to the TCA even in a mature biofilm condition (Supplementary Figure S1). Moreover, 34 additional mutants could grow better than the reference strain even when a mature biofilm was challenged (Figure 2d). According to KEGG, the more representative categories in this condition were again ribosome biogenesis and cell cycle/meiosis control. The enriched categories identified with GO Process Slim Mapper (Supplementary Table S3) were organelle organization, transport, RNA metabolic process, cellular protein modification, response to chemical or stress, and cell cycle.

### 3.3. Analysis of Sensitive and Tolerant Mutants to More Than One Drug

If the mutants identified are involved in a general cellular response to stress, then the phenotype would be displayed not only in the presence of NOR, but also when other antimicrobials are present in medium. Mutants showing general sensitivity or tolerance to drugs are reported in Supplementary Table S5 and Figure 1a–c. As displayed in Supplementary Table S5, 26 strains were sensitive to all the treatments during biofilm formation, while four demonstrated the same phenotype at the beginning of the colonization as well as in a mature biofilm. Among those, most of the deleted open reading frames (ORFs) have been poorly characterized and the role attributed is often only predicted. The better characterized deleted genes are involved in oxidative stress responses (*GND1*, *MPS1*), regulation of filamentous growth and stress responses (*VPS4*, *VPS23*, *CDC9*, *ENP1*), control of transcription and translation (*TFB1*, *MED8*, *SSU72*, *NEP1*, *PAB1*), and transport (*LYP1*, orf19.304, *NAG4*). Overall, 25 mutants were sensitive to NOR, AMB, and CAS, while five were sensitive to all four drugs tested (Figure 1c). Genes that mutated gave sensitivity even to FLU were as follows: *MNN2* (alpha-1, 2-mannosyltransferase with a role in cell wall integrity), *FLC3* (involved in heme uptake and putative FAD transporter), *MED8* (ortholog(s) have RNA polymerase II core promoter proximal region sequence-specific DNA binding), orf19.3852 (catalytic activity and role in carbohydrate metabolic process), and orf19.3037 (putative poly (A)-binding protein) [22,23].

During the formation of a biofilm, only three mutants were tolerant to the antimicrobials used, while nine were tolerant even when a mature biofilm was challenged (Table S5). These 12 mutated genetic functions are poorly characterized, and the ones with a known activity correspond to *PLB1*, a phospholipase B involved in virulence, and *SHA3* and *SCH9*, involved in stress responses and in filamentous growth; among these, mutants’ tolerance to all four drugs was displayed by six strains (Figure 1d), where the deleted genes were *PGA12*, *CPR52*, *RTS2*, *PAA11*, orf19.305, and orf19.1180.

### 3.4. Determination of Oxidative and Nitrosative Stress Induced by Different Drugs

**Figure 3:**
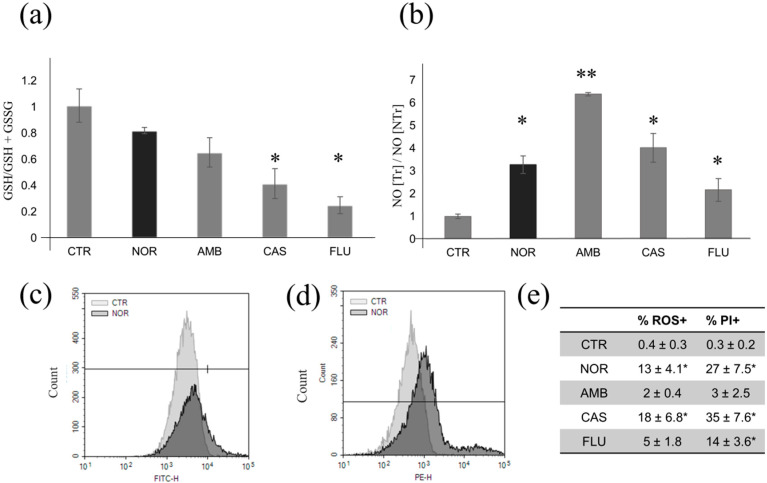
Oxidative stress in *Candida albicans* grown in the presence of NOR, AMB, CAS, or FLU: (**a**) effects of the drugs on glutathione oxidation state after 24 h of treatment; the results can be compared to those in the untreated control (CTR); (**b**) levels of nitric oxide (NO) of cells grown in the presence of the drugs for 24 h versus the CTR; (**c**) comparison of the distribution of cells reactive oxygen species (ROS+) (FITC (Fluorescein isothiocyanate) channel) or propidium iodide (PI+) (**d**) (PE channel) in the CTR or in cells treated with NOR; (**e**) table summarizing the flow cytometry data obtained for cells grown in the presence of NOR, AMB, CAS, or FLU. Marks on the histogram and in the table indicate that data, compared with the control using a t-test, were significantly different (* if *p* < 0.05 and ** if *p* < 0.01).

Environmental stress such as the exposure to xenobiotics could determine an oxidative stress with the production of reactive oxygen species (ROS). To test the level of oxidative stress induced by NOR or by the other antifungals, the glutathione oxidation state (the ratio of GSH/(GSH + GSSG) where GSH is reduced glutathione and GSSG is oxidized glutathione), the relative concentration of nitric oxide (NO), and the amounts of ROS were tested (Figure 3a). The oxidized glutathione was present when cells were treated with CAS and FLU, while an increase in NO was observed in all conditions (Figure 3b). Exposure to NOR and CAS increased the ROS content per cell, as well as the percentage of dead cells (Figure 3c–e).

### 3.5. DNA Damage

Data reported in the previous paragraphs suggest that mutants in mismatch and DNA repair better tolerate a treatment with NOR, suggesting that treatment with the TCA is related to DNA damage. The Ames test with *S. typhimurium* TA98 and TA100 was performed and the data in Supplementary Table S6 showed that the highest concentrations of NOR tested (600 µM) induced an increase in base shift revertants, but a low increase in frameshifts. At concentrations closer to those used in the experiments described above, a real mutagenic effect was not evident. The addition of S9 liver fraction reduced the mutation rate frequency to that of the control.

### 3.6. Expression Analysis of Transporter Genes

**Figure 4:**
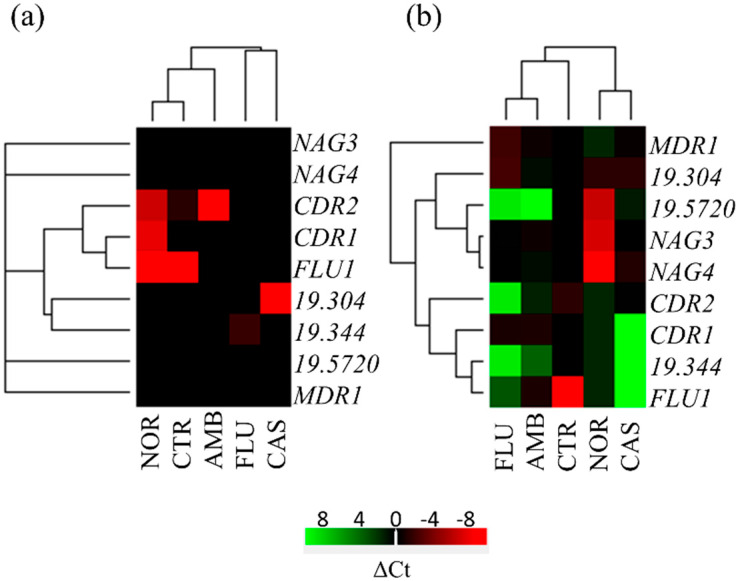
Heat-maps showing the expression changes in a mature biofilm of known drugs transporters (*MDR1*, *CDR1*, *CDR2*), or of the least characterized ones (*FLU1*, *19.5720*, *NAG3*, *NAG4*, *19.304*, *19.344*) after 4 h (**a**) or 24 h (**b**) of treatment with AMB, CAS, FLU, and NOR. A sample was taken after the 48 h of growth and before adding the drugs, and represents the CTR, which was selected as the reference sample. The heat-map was made with the DataAssist software (Thermo Fisher Scientific Inc., Waltham, MA, USA) and global normalization was applied. Distance between samples was calculated using Pearson’s correlation and average linkage was employed for clustering. Color legend is presented below the heat maps; the green color is for downregulation of gene expression and the red color is for gene upregulation.

Mdr1, Cdr1, and Cdr2 are plasma membrane multidrug efflux pumps often overexpressed in antifungal-resistant clinical isolates. In the HIP screening, transporter genes whose deletion increased sensitivity to all drugs tested (*19.304*, *NAG4*, *19.3444*) or specifically only to NOR (*FLU1*, *NAG3*, *19.5720*) were identified. To examine their involvement in xenobiotic detoxification, the expression of these genes was tested in the presence of the different drugs, along with the expression of the well-known *MDR1*, *CDR1*, and *CDR2*. Basal expression of the selected genes was absent in a mature biofilm grown for 48 h (Figure 4). Incubation with any of the drugs for 4 h induced the expression of only a few genes, but not in all of the conditions tested (Figure 4a and Supplementary Figure S2). *FLU1* is repressed in the presence of NOR, CAS, and FLU; *CDR1* is induced by NOR; *CDR2* is induced by NOR and AMB; and *19.304* by CAS. There were more changes after 24 h (Figure 4b and Supplementary Figure S2). The expression of *CDR1* is induced by AMB and FLU; the expression of *CDR2* increased when AMB or CAS is present in the medium; and *MDR1* is upregulated when AMB, CAS, and FLU are present. *NAG3*, *NAG4*, and *19.5720* are strongly induced by NOR, while *CDR2* and *19.304* are upregulated in the presence of AMB and CAS. *FLU1* is repressed by CAS, FLU, and NOR, while *19.304* is induced by the same antimicrobials.

## 4. Discussion

*Candida* spp. causes morbidity and mortality in humans; in 2017, almost 35,000 people were hospitalized and 1700 died in the United States as a result of *Candida*-related infections [24]. Commonly used drugs to eliminate *Candida* spp. infections include the polyene AMB and the azole FLU, both targeting the ergosterol pathway, and CAS, inhibiting the synthesis of β-glucans of the cell wall [25]. AMB and CAS can both inhibit biofilm formation. While the latter is also able to decrease dispersion [26], FLU can kill cells, but is not able to reduce biofilm formation [27,28]. Biofilm constitutes a significant health problem because, within this structure, antimicrobial resistance is easily developed. This phenotype is often associated with overexpression of efflux pumps (Cdr1, Mdr1, and Mdr2) and modifications of sterol composition [29]. It is suggested that drug resistance could be bypassed by a two-drug combination that work synergistically, a possibility that could also reduce host toxicity [30]. By applying wet and dry lab approaches, also involving computational and artificial intelligence [3], scientists are trying to identify new and effective antimicrobial drugs by exploring the possibility that existing drugs could display new therapeutic functions. Following this approach, researchers found that miltofosine and toremifene (originally considered an antitumor agent), 2-adamantanamine (an anti-influenza A virus drug), and the antidepressant sertraline have antimicrobial properties that are now tested on in vivo models [9,10,27,31,32]. Another antidepressant with antimicrobial properties that was shown to be active against *Candida albicans* is NOR [11], but little is known about its potential target(s) and molecular effects. This paper examines these aspects by combining a chemogenomic profiling with bioinformatic analysis, physiological tests, and gene expression data. Several *Candida albicans* genes still need to be characterized, and this is also reflected in this study as gene function is often reported as putative, predicted, or inferred (based on known orthologues from *Saccharomyces cerevisiae*). The retrieved data seem to suggest that mitochondria are among the targets of NOR, a characteristic common to other central nervous system drugs [33]. Mutants in oxidative phosphorylation and fatty acid metabolism are more sensitive to the treatment with NOR. The synthesis of ROS was increased upon treatment with NOR. Oxidative stress has been suggested to induce cell damage and cell death [34]; indeed, the number of dead cells also increased in the presented experiments (see PI+ cells). Overall, this is an effect that may have clinical relevance because it is known that oxidative stress reduces microbial pathogenicity by enhanced killing by macrophages [35], as shown for *A. fumigatus* [36]. Beside NOR, the most effective drug in the conditions tested was CAS, where the glutathione oxidation state was lower, while ROS, NO, and PI+ cells were higher, confirming previous data showing the capacity of CAS to induce ROS and cell death [37]. In our case, the presence of NOR also induces NO production. In higher organisms, NO is involved in cell signaling and maintenance of homeostasis, but high levels of this metabolite lead to chemical stress, possibly also contributing to the induction and progression of diseases [38,39]. The situation in yeast is far less clear. Recently, in *Candida albicans*, a nitric oxide synthase pathway-like has been investigated [40], while in *Saccharomyces cerevisiae*, the flavoprotein Tah18 is involved in NO synthesis, and increases in this enzyme activity confer high-temperature stress tolerance to yeast [41]. TAH18 has an ortholog in *C. albicans* (orf19.2040), but its activity has yet to be confirmed. Therefore, in yeast, and especially in *C. albicans*, the role of NO in cell physiology, the pathways involved in its synthesis, and the targets of nitrosative stress still need to be clarified.

Targets of NOR are also ribosome biogenesis and machinery, RNA binding, and the RNA splicing apparatus. This result suggests a miss-regulation of protein synthesis. Similarly, exposure of a mature biofilm to AMB or CAS changes the expression of ribosome biogenesis-related genes [42]. A different concentration of proteins involved in mismatch repair and DNA replication makes the yeast more tolerant to NOR, a phenotype not linked to DNA damage, as the latter was observed only at drug concentrations higher than those used in this work, and to that employed to treat depression syndromes [43]. It is interesting to note that many of the targets are regulated by the transcription factor Hap43, a protein with pleiotropic functions (i.e., iron homeostasis, chromosome biogenesis, and filamentation [44]).

To identify NOR specific and aspecific targets, sensitive and tolerant mutants identified in the first screenings were also checked for sensitivity and tolerance to other drugs. Thirty mutants were sensitive to all the tested drugs (except for FLU, which had a small impact in the test conditions) during biofilm formation or during its maintenance, while 12 mutants were more tolerant than the control. They belong to the GO categories: ribosome biogenesis, RNA splicing, and mRNA processing, indicating that these functions are general targets of antimicrobials drugs [42]. Interestingly, antimicrobial molecules specifically targeting these structures are now under investigation [45]. The genes listed (in Supplementary Data) might be identified as *C. albicans* specific MDR genes [46], as very little overlap is displayed between these elements and the MDR genes described in *S. cerevisiae* [46,47].

The drug efflux pumps *MDR1*, *CDR1*, and *CDR2* are often described as MDR genes. Indeed, expression of these ATP binding cassette transporters (ABC) is often higher in resistant clinical isolates [46,48]. A lower concentration of the proteins encoded by these genes does not affect the sensitivity to NOR, but a decreased level of the least characterized Flu1, Nag3, and product of orf19.5720 transporters makes *C. albicans* more sensitive to NOR. Lower levels of orf19.304 product, a putative fungal specific MDR protein, and Nag4 make the yeast more sensitive to NOR, AMB, and CAS. Overall, the expression of these transporters in a mature biofilm is almost absent and changes only slightly after 4 h of treatment with antimicrobials, instead, after 24 h, all genes are upregulated, but their expression is different between treatments. Expression of orf19.5720, *NAG3*, and *NAG4* increases especially in the presence of NOR, whereas the expressions of *MDR1*, *CDR1*, and *CDR2* were unaffected by the presence of NOR. This shows how the detox-response in *Candida albicans* to this TCA is specific and different from the response to other antifungals. In addition, this suggests that product of orf19.5720, Nag3, and Nag4 may also be relevant for acquired antimicrobial resistance. This hypothesis is supported by the observation that the combination of deletion in the three common ABC transporters does not eliminate drug resistance [29] and suggests that other mechanisms could be involved.

## 5. Conclusions

Considering the above in *C. albicans*: (I) the antimicrobial activity of NOR is synergic with other commercial drugs [11], (II) this TCA has different targets than those of known antimicrobials, and (III) NOR elicits different detox strategies. These data suggest NOR could be repositioned as a “new” antimicrobial drug ready to be tested on in vivo models to investigate its potential clinical application.

## Supplementary Materials

The following are available online at https://www.mdpi.com/2076-2607/8/5/742/s1, Figure S1: GP of tolerant or sensitive mutants versus wild type upon tetracycline addition to a mature biofilm; Figure S2: Raw data of gene expression data; Table S1: List of primers used in qRT-PCR; Table S2: List of mutants sensitive to NOR; Table S3: GO analysis with Slim Mapper; Table S4: List of mutants sensitive to NOR; Table S5: List of mutants sensitive or tolerant to NOR, AMB, and CAS; Table S6: Ames mutagenicity test with NOR.
