# Supplementary material for: Known Antimicrobials Versus Nortriptyline in Candida albicans: Repositioning an Old Drug for New Targets
Source: Microorganisms. 2020 May 15;8(5):742. doi: 10.3390/microorganisms8050742 (PMC7284794; doi:10.3390/microorganisms8050742)

Table S1: List of primers used in qRT-PCR

Gene target	Primer Fw	Primer Rv
RDN18	CACGACGGAGTTTCACAAGA	CGATGGAAGTTTGAGGCAAT
MDR1	GCCCATTGGTTTTCAGTCCG	TGGCCAAACAAGGACTAGCA
CDR1	TGTTGGGTTGGTCTCGATGG	CCCCACTGCAGTACACACTT
CDR2	TCCGAGGTGGAGCACTTTTC	TGCATCAGCAGATGGACGAT
FLU1	CGAGAAGAGCCACCAGAAGT	TCATCGGGACCATCAAAGGC
NAG3	GCTGGTCAGTTAGGGCCATT	GGTCCAGTCCACTAAACGCA
NAG4	ACGCTAGAAGCTTGGTGTCC	CTTCCAAGGGTCGGCTTCAT
19.304	TGCCCTCTTGATACCGTGTG	GTGCAATCAAGGCCGCTATG
19.5720	TAATGGGGGCAGCACAAAGT	AACCCTCCAATTGACCCACC
19.3444	ATTACGCCGGTGATGATGCT	GATGGGCCACCCATTTCTGA

Table S2: List of mutants sensitive to NOR.

The list was divided in two sections: (I) mutants sensitive to NOR during formation of biofilm and (II) mutants that results sensitive to the drug even in a mature biofilm.

MUTANTS SENSITIVE TO NORTRYPTILINE DURING BIOFILM FORMATION

Inactivated gene identifier	Standard name of inactivated gene	Function
orf19.6524	*TOM40*	Protein involved in mitochondrial protein import;
orf19.5407	*SOF1*	Putative protein with a predicted role in 40S ribosomal subunit biogenesis;
orf19.3575	*CDC19*	Pyruvate kinase at yeast cell surface; Gcn4/Hog1/GlcNAc regulated;
orf19.6045	*PSD1*	Putative phosphatidylserine decarboxylase; involved in phosphatidylethanolamine;
orf19.5455	*VTC1*	Ortholog(s) have mRNA binding, small GTPase regulator activity;
orf19.7257	*MLH3*	Ortholog(s) have role in mismatch repair;
orf19.4326	*SNU66*	Ortholog(s) have role in mRNA cis splicing, via spliceosome;
orf19.4312	*SPT8*	Ortholog(s) have TBP-class protein binding, transcription cofactor activity;
orf19.2158	*NAG3*	Putative MFS transporter; similar to Nag4;
orf19.6595	*RTA4*	Protein similar to *S. cerevisiae* Rsb1p, involved in fatty acid transport;
orf19.1531	*RNA14*	Ortholog(s) have RNA binding, mRNA cleavage, mRNA polyadenylation;
orf19.7388	*PBS2*	MAPK kinase; role in osmotic and oxidative stress responses, oxidative stress adaptation;
orf19.2059	*EEP2*	Protein with homology to magnesium-dependent endonucleases and phosphatases;
orf19.6531	*NUC2*	Putative NADH-ubiquinone oxidoreductase;
orf19.7071	*FGR2*	Protein similar to phosphate transporters;

MUTANTS SENSITIVE TO NORTRYPTILINE DURING BIOFILM FORMATION AND IN A MATURE BIOFILM

orf19.606		Protein of unknown function;
orf19.275	*POP5*	Ortholog(s) have ribonuclease MRP activity, ribonuclease P activity;
orf19.1975	*DIB1*	Ortholog(s) have role in mRNA splicing, via spliceosome;
orf19.5379	*ERG4*	Protein similar to sterol C-24 reductase; shows Mob2p-dependent hyphal regulation;
orf19.1649	*RNA1*	Putative GTPase-activating protein; protein level decreases in stationary phase cultures;
orf19.5465	*YJU2*	Ortholog(s) have first spliceosomal transesterification activity;
orf19.3087	*UBI3*	Fusion of ubiquitin with the S34 protein of the small ribosomal subunit;
orf19.7645	*BPL1*	Biotin protein ligase;
orf19.7567		Protein of unknown function;
orf19.1125	*HWH4*	Predicted hydrolase;
orf19.7523	*MKC1*	MAP kinase; phosphorylated on membrane perturbation, or cell wall stress;
orf19.5949	*FAS2*	Alpha subunit of fatty-acid synthase;
orf19.6034	*TUB2*	Beta-tubulin; functional homolog of ScTub2p;
orf19.5964	*ARF2*	Putative ADP-ribosylation factor;
orf19.2839	*CIRT4B*	Cirt family transposase;
orf19.5953	*SFP1*	C2H2 transcription factor;
orf19.4370		Protein of unknown function;
orf19.121	*ARC18*	Putative ARP2/3 complex subunit;
orf19.3237	*UFD*	Ortholog(s) have cytosol, mitochondrion, nucleus localization;
orf19.5630	*APA2*	Putative ATP adenylyltransferase II; regulated by Gcn4;

orf19.144	*SNU114*	Protein similar to *S. cerevisiae* Snu114p, an RNA helicase involved in splicing;
orf19.3465	*RPL10A*	Predicted ribosomal protein;
orf19.7016	*PHM5*	Ortholog(s) have endopolyphosphatase activity;
orf19.3545	*CDC15*	Ortholog(s) have protein serine/threonine kinase activity;
orf19.6312	*RPS3*	Ribosomal protein S3;
orf19.2574	*IFU4*	Ortholog(s) have Golgi apparatus localization;
orf19.5205		Protein of unknown function;
orf19.6175		Putative 35S rRNA processing protein;
orf19.5720		Predicted membrane transporter, member of the monocarboxylate porter (MCP) family, major facilitator superfamily (MFS);
orf19.6195	*RNH70*	Ortholog(s) have 3'-5'-exoribonuclease activity;
orf19.500		Ortholog(s) have tRNA (adenine-N1-)-methyltransferase activity, role in tRNA methylation and nucleus, tRNA (m1A) methyltransferase complex localization;
orf19.3409	*SEC12*	Putative guanyl-nucleotide exchange factor; induced in high iron;
orf19.3010	*LAB2*	Putative lipoyl ligase;
orf19.2312	*FRE3*	Protein similar to ferric reductase Fre10p;
orf19.6007		Predicted fatty acid acyl transferase-related protein domain;
orf19.3327	*TRM2*	Putative tRNA methyltransferase; repressed by prostaglandins;
orf19.6577	*FLU1*	Multidrug efflux pump of the plasma membrane; MDR family member;
orf19.5532		Protein of unknown function;

Table S3: GO analysis with Slim Mapper.

The table was created using the CGD database (www.candidagenome.org) of the sensitive and tolerant mutants identified in the screening. Top seven GO categories are reported for GO-Function and GO-Process.

**GO analysis with Slim Mapper of sensitive mutants**			
Function		*Process*	
GO term	Cluster frequency	GO term	Cluster frequency
Molecular function unknown	26.9 %	RNA metabolic process	25.0 %
Transferase activity	19.2 %	Transport	23.1 %
Hydrolase activity	16.4 %	Organelle organization	21.1 %
RNA binding	13.5 %	Biological process unknown	17.3 %
Protein binding	9.6 %	Ribosome biogenesis	15.4 %
Transporter activity	9.6 %	Cellular protein modification	15.4%
Oxidoreductase activity	7.7%	Response to chemicals	13.5 %
**GO analysis with Slim Mapper of tolerant mutants**			
Function		*Process*	
GO term	Cluster frequency	GO term	Cluster frequency
Transferase activity	20.3 %	Organelle organization	25.3 %
Molecular function unknown	16.5 %	Regulation of biological process	24.1 %
Protein binding	16.5%	Transport	19.0 %
Hydrolase activity	13.9 %	RNA metabolic process	17.7 %
DNA binding	10.1 %	Cellular protein modification	13.9 %
Transporter activity	10.1 %	Response to chemicals and stress	12.7 %
Oxidoreductase activity	7.6 %	Cell cycle	11.4 %

Table S4: List of mutants tolerant to NOR.

The list was divided in two sections: (I) mutants tolerant to NOR during formation of biofilm and (II) mutants that results tolerant to the drug even in a mature biofilm.

MUTANTS TOLERANT TO NORTRYPTILINE DURING BIOFILM FORMATION

Inactivated gene identifier	Standard name of inactivated gene	Function
orf19.5861	*KRE9*	Protein of beta-1,6-glucan biosynthesis; required for serum-induced hyphal growth;
orf19.2313		Putative ortholog of *S. cerevisiae* Pex32 a peroxisomal integral membrane protein
orf19.2029	*RFC5*	Putative heteropentameric replication factor C subunit
orf19.7492	*SWC4*	Subunit of the NuA4 histone acetyltransferase complex
orf19.4205.1	*SME1*	Ortholog(s) have role in mRNA splicing,
orf19.793	*CAK1*	Monomeric CDK-activating kinase;
orf19.3348		Ortholog(s) have structural constituent of ribosome activity;
orf19.339	*NDE1*	Putative NADH dehydrogenase; may act alternatively to complex I in respiration;
orf19.2939	*FMP32*	Ortholog(s) have mitochondrion localization
orf19.6039	*SED5*	Ortholog(s) have SNAP receptor activity, role in ER to Golgi vesicle-mediated transport;
orf19.6044	*MOB2*	Mob1/phocein domain protein of RAM signaling network;
orf19.2008		Ortholog(s) have S-methyl-5-thioribose-1-phosphate isomerase activity;
orf19.6900	*MDM12*	Ortholog(s) have role in mitochondrial genome maintenance;
orf19.403	*PCL2*	Cyclin homolog; reduced expression observed upon depletion of Cln3;
orf19.1285		Plasma membrane-localized protein of unknown function
orf19.6727	*RIT1*	Putative initiator tRNA methionine ribosyltransferase;
orf19.4771	*MNN47*	Protein of unknown function;
orf19.5103	*PMU5*	Protein with a predicted phosphoglycerate mutase family domain;
orf19.6956	*DAL9*	Putative allantoate permease; fungal-specific (no human or murine homolog)
orf19.3458		Ortholog(s) have role in late endosome to vacuole transport via multivesicular body sorting pathway, protein targeting to vacuole and Golgi apparatus;
orf19.2309	*PET127*	Protein with a predicted role in 5'-end processing of mitochondrial RNAs;
orf19.4187	*MMM1*	Ortholog(s) have role in mitochondrial genome maintenance;
orf19.6464		Protein of unknown function; induced upon adherence to polystyrene;
orf19.6358	*MMS2*	Ortholog(s) have protein homodimerization activity, ubiquitin-protein ligase activity;
orf19.4551	*CTN1*	Carnitine acetyl transferase;
orf19.4473	*SPC19*	Essential subunit of the Dam1 (DASH) complex, which acts in chromosome segregation;
orf19.5530	*NAB3*	Putative nuclear polyadenylated RNA-binding protein;
orf19.2114	*URO99*	Predicted uricase; ortholog of *S. pombe* SPCC1223.09;
orf19.3700	*TOM70*	Ortholog(s) have mitochondrion targeting sequence binding;
orf19.10	*ALK8*	Alkane-inducible cytochrome P450;
orf19.2112	*PRP18*	snRNP U5 splicing factor component;
orf19.4950	*AKR1*	Ankyrin-repeat protein;
orf19.3699	*TEP1*	Putative protein phosphatase of the PTP family (tyrosine-specific);
orf19.4592	*HSX11*	UDP-glucose:ceramide glucosyltransferase;
orf19.1686	*MPA43*	Ortholog(s) have mitochondrion localization;
orf19.684	*PCF11*	Putative transcription factor with zinc finger DNA-binding motif;
orf19.6749	*KRS1*	Putative tRNA-Lys synthetase; repressed upon phagocytosis by murine macrophages;

orf19.2146	*HAT2*	Putative Hat1-Hat2 histone acetyltransferase complex subunit;
orf19.496		Ortholog(s) have DNA-dependent ATPase activity, dinucleotide insertion or deletion binding, guanine/thymine mispair binding activity, role in mismatch repair;
orf19.3035	*CHD1*	Ortholog(s) have ATP-dependent DNA helicase activity, chromatin DNA binding;
orf19.5978		Has domain(s) with predicted nucleotide binding, oxidoreductase activity, role in oxidation-reduction process;
orf19.1799	*GAP5*	Putative general amino acid permease; fungal-specific (no human or murine homolog)
orf19.183	*HIS3*	Imidazoleglycerol-phosphate dehydratase, enzyme of histidine biosynthesis; (no human or murine homolog)
orf19.4351	*PRP13*	Putative integral inner mitochondrial membrane protein with similarity to exonucleases;
**MUTANTS SENSITIVE TO NORTRYPTILINE DURING BIOFILM FORMATION AND IN A MATURE BIOFILM**		
orf19.6512	*EXO70*	Predicted subunit of the exocyst complex, involved in exocytosis;
orf19.5551	*MIF2*	Centromere-associated protein; similar to CENP-C proteins;
orf19.5815	*SCT2*	Putative glycerol-3-phosphate acyltransferase
orf19.3966	*CRH12*	CRH family cell wall protein;
orf19.4221	*ORC4*	Phosphorylated protein subunit of the origin recognition complex (ORC);
orf19.4628		Putative cleavage and polyadenylation factor;
orf19.6981		Ortholog of *S. cerevisiae* : YKR051W;
orf19.3029	*EHD3*	Predicted 3-hydroxyisobutyryl-CoA hydrolase;
orf19.1130	*IFI1*	Protein of unknown function;
orf19.216		Protein with a metallo-dependent phosphatase domain;
orf19.4546	*HOL4*	Putative ion transporter; alkaline induced by Rim101;
orf19.4060	*ARO4*	3-deoxy-D-arabinoheptulosonate-7-phosphate synthase;
orf19.5894		Ortholog of *S. cerevisiae* : YEL023C;
orf19.2084	*CDH1*	Protein involved in regulation of mitosis;
orf19.1264	*CFL2*	Oxidoreductase;
orf19.4967	*COX19*	Putative cytochrome c oxidase assembly protein;
orf19.5873	*POL1*	Putative DNA directed DNA polymerase alpha;
orf19.6081	*PHR2*	Glycosidase;
orf19.5004	*RAD54*	Putative DNA-dependent ATPase involved in DNA repair;
orf19.4240	*COS162*	Ortholog(s) have role in GPI anchor biosynthetic process;
orf19.4410	*ALG1*	mannosyltransferase involved in N-linked protein glycosylation;
orf19.2762	*AHP1*	Alkyl hydroperoxide reductase;
orf19.4928	*SEC2*	Guanyl-nucleotide exchange factor for the small G-protein Sec4;
orf19.6562	*RNH35*	Putative ribonuclease H2 catalytic subunit;
orf19.3444	*SGE13*	Predicted membrane transporter, member of the drug:proton antiporter;
orf19.2104	*JAC1*	Ortholog(s) have chaperone binding activity, role in aerobic respiration;
orf19.661	*KRR1*	Putative nucleolar protein;
orf19.401	*TCP1*	Chaperonin-containing T-complex subunit;
orf19.2830	*RRP9*	Ribosomal protein;
orf19.5492	*YHC1*	Ortholog(s) have mRNA binding activity, role in mRNA splicing;
orf19.2117	*LEU5*	Putative mitochondrial carrier protein;
orf19.5858	*EGD2*	Nascent polypeptide associated complex protein alpha subunit;
orf19.7516	*NGS1*	Putative glycoside hydrolase;
orf19.1946		Similar to an aldose 1-epimerase-related protein;

Table S5: **List of mutants sensitive or tolerant to NOR, AMB and CAS.**

The list was divided in four sections: (I) mutants sensitive to drugs during formation of biofilm and (II) mutants that results sensitive to the antibiotics tested even in a mature biofilm; and (III) mutants tolerant to NOR, AMB and CAS during biofilm formation and (IV) tolerant in a mature biofilm.

Inactivated gene identifier	Standard name of inactivated gene	Function
**MUTANTS SENSITIVE DURING BIOFILM FORMATION**		
orf19.4339	*VPS4*	AAA-ATPase involved in transport from MVB to the vacuole and ESCRT-III complex disassembly;
orf19.2347	*MNN2*	Alpha-1,2-mannosyltransferase; similar to *S. cerevisiae* Mnn2;
orf19.3852		Has domain(s) with predicted catalytic activity
orf19.3720		Ortholog of *Candida tenuis* NRRL Y-1498;
orf19.5297	*TFB1*	Ortholog(s) have phosphatidylinositol-3-phosphate binding;
orf19.4497	*MED8*	Ortholog(s) have RNA polymerase II core promoter proximal region sequence-specific DNA binding;
orf19.1533		Possible vacuolar protein;
orf19.2514		Predicted methyltransferase;
orf19.2198	*FLC3*	Protein involved in heme uptake;
orf19.851	*MNN42*	Fungal-type cell wall polysaccharide biosynthetic process
orf19.7140	*CMT1*	Putative catechol o-methyltransferase;
orf19.7314	*CDG1*	Putative cysteine dioxygenases;
orf19.3230	*BOI2*	Putative SH3-domain-containing protein;
orf19.304		Putative transporter similar to MDR proteins; fungal-specific;
orf19.2160	*NAG4*	Putative transporter;
orf19.5024	*GND1*	6-phosphogluconate dehydrogenase;
orf19.2251	*AAH1*	Adenine deaminase; purine salvage and nitrogen catabolism;
orf19.2343	*VPS23*	ESCRT I protein sorting complex protein;
orf19.4726	*FRQ1*	Ortholog(s) have calcium ion binding, enzyme activator activity;
orf19.2402	*SSU72*	Ortholog(s) have CTD phosphatase activity, protein tyrosine phosphatase activity;
orf19.6155	*CDC9*	Ortholog(s) have DNA ligase (ATP) activity;
orf19.665	*NEP1*	Ortholog(s) have protein homodimerization activity, rRNA binding activity;
orf19.6143	*FAO1*	Predicted long-chain-alcohol oxidase;
orf19.5507	*ENP1*	Protein required for pre-rRNA processing and 40S ribosomal subunit synthesis;
orf19.1224	*FRP3*	Putative ammonium transporter;
orf19.4826	*IDH1*	Putative mitochondrial NAD-isocitrate dehydrogenase subunit 1;
**MUTANTS SENSITIVE DURING BIOFILM FORMATION AND IN A MATURE BIOFILM**		
orf19.651	*LYP1*	Putative permease
orf19.3037	*PAB1*	Putative poly(A)-binding protein;
orf19.7293	*MPS1*	Monopolar spindle protein, a putative kinase;
orf19.2386		Ortholog(s) have role in endonucleolytic cleavage in 5'-ETS of tricistronic rRNA transcript (SSU-rRNA, 5.8S rRNA and LSU-rRNA);
**MUTANTS TOLERANT DURING BIOFILM FORMATION**		
orf19.3669	*SHA3*	Putative ser/thr kinase involved in glucose transport;
orf19.2575	*IFU3*	Putative S-adenosylmethionine-dependent methyltransferase;
orf19.689	*PLB1*	Phospholipase B; host cell penetration and virulence in mouse systemic infection;

MUTANTS TOLERANT DURING BIOFILM FORMATION AND IN A MATURE BIOFILM		
orf19.829	*SCH9*	Protein kinase; involved in growth control;
orf19.1305		Ortholog(s) have tRNA (guanine) methyltransferase activity, role in tRNA methylation and mitochondrial matrix;
orf19.7597	*PGA12*	Putative GPI-anchored protein;
orf19.4229	*DDP1*	Putative polyphosphate phosphatase;
orf19.1180		Putative 2-aminoadipate transaminase;
orf19.6990	*CPR52*	Putative peptidyl-prolyl cis-trans isomerase;
orf19.3817	*RTS2*	Ortholog(s) have activity in cytoplasm;
orf19.4006		Ortholog(s) have activity in cytosol;
orf19.7269	*PAA11*	Putative polyamine acetyltransferase;

Table S6: Ames mutagenicity test with NOR

S.typhimurium TA98 and TA100 were used in this test. For each strain two doses of NOR were used along with a positive control (2-nitrofluorene for TA98 and 2-aminoantracene for TA100). The table reports the concentrations used in the test, the number of revertant per plate of the treated versus the not treated and the fold-increase of the mutations. The data are reported only without the addition of S9 to the mix, as adding this component lowered the mutation rate to level similar to the control.

	Type of point mutations	Molecule	Dose	Rev./plate (T/NT)	Fold-increase
**TA98**	frameshift				
		2-nitrofluorene	2 µg	66/36	1.8
		nortryptiline	60 µg (600 µM)	378/36	**10.5**
		nortryptiline	12µg (120 µM)	32/36	0.9
**TA100**	base substitution				
		2-aminoantracene	1µg	>2000/108	>20
		nortryptiline	60 µg (600 µM)	200/108	**1.9**
		nortryptiline	12µg (120 µM)	128/108	1.2

Figure S1: GP of tolerant or sensitive mutants versus wild type upon tetracycline addition to a mature biofilm.

Three tolerant or sensitive mutants displaying the mentioned GP only during biofilm formation were selected. Strains were either challenged during biofilm formation (BF) by NOR (BF + NOR), or allowed to form a mature biofilm (MF) (as described in the Material and Methods section of the main text) and then challenged with NOR for 48 hours (MB + NOR) to confirm the GPs; or with NOR + tetracycline (as described in Romer *et al*., 2003) for 48 hours (MB + NOR + TET). The results showed that during biofilm formation, sensitive mutants have a GPs reduced of almost 2.5 folds compared the WT, while tolerant mutants display a GPs at least 4 times higher than the WT, all behave similarly to the reference strains when a mature biofilm is challenged only with NOR (the ratio close to 1). Adding tetracycline, to turn off the expression of also the second copy of the gene, makes the strain sensitive or tolerant also as a mature biofilm.

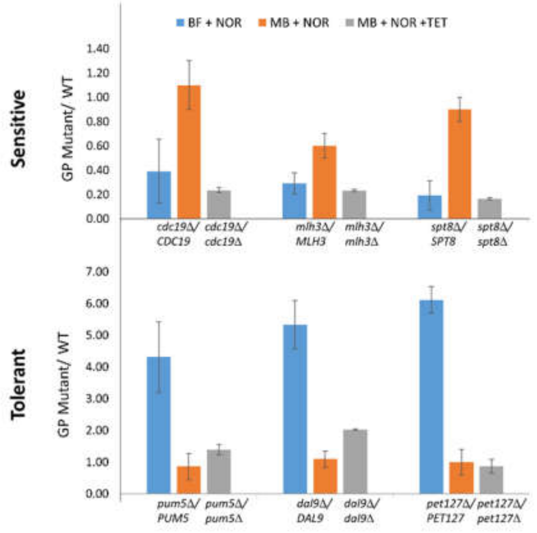

Figure S2: Raw data of gene expression data.

The graphs report the raw Ct data obtained after the RealTime-qPCR. The data show the expression changes in a mature biofilm of known drugs transporters (*MDR1, CDR1, CDR2*), or of the least characterized ones (*FLU1*, 19.5720, *NAG3, NAG4*, 19.304,19.344) after 4 hours (a) or 24 hours (b) of treatment with AMB, CAS, FLU and NOR. A sample was taken after the 48 hours of growth and before adding the drugs and it represents the CTR. Data were overall analysed with DataAssist software (Thermo Fisher Scientific Inc., MA USA) as described in the main text.

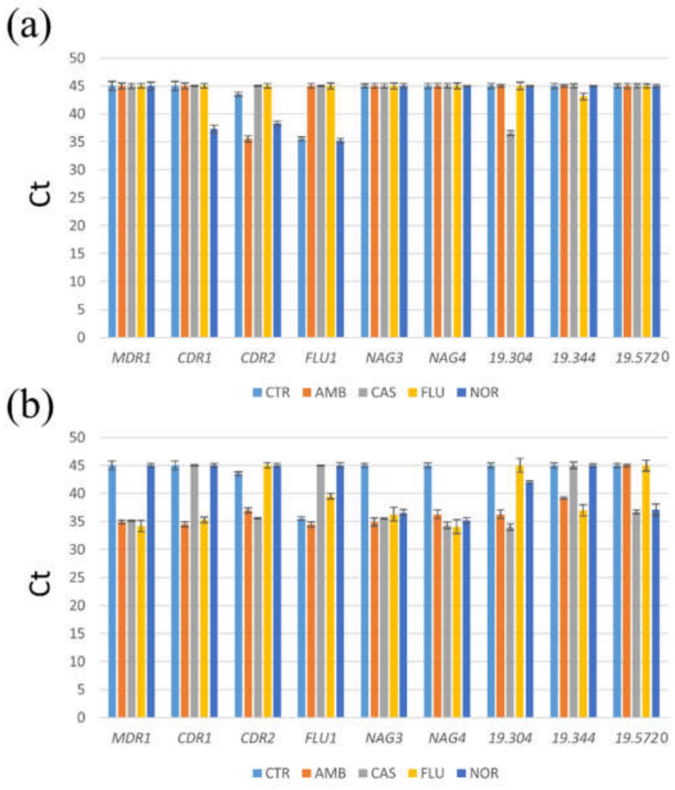

Supplement: Supplementary file 1 [file microorganisms-08-00742-s001.pdf]
